# Study of the association between gait variability and physical activity

**DOI:** 10.1186/s11556-017-0188-0

**Published:** 2017-11-15

**Authors:** Daniela Ciprandi, Filippo Bertozzi, Matteo Zago, Claudia Lucia Pimenta Ferreira, Giuseppe Boari, Chiarella Sforza, Christel Galvani

**Affiliations:** 1 Movement Analysis Laboratory, Department of Biomedical Sciences for Health, Università degli Studi di Milano, via Mangiagalli 31, I-20133 Milan, Italy; 2 Exercise and Sport Science Degree Course, Faculties of Education and Medicine and Surgery, Università Cattolica del Sacro Cuore, Vle Suzzani 279, I-20162 Milan, Italy; 3 Movlab, Movement and Posture Analysis Lab, Department of Electronics, Information and Bioengineering, Politecnico di Milano, via Golgi 39, I-20133 Milan, Italy; 4 Department of statistical science, Faculty of economics, Università Cattolica del Sacro Cuore, Largo Gemelli 1, I-20123 Milan, Italy; 5 Applied Exercise Physiology Laboratory, Department of Psychology, Università Cattolica del Sacro Cuore, Vle Suzzani 279, I-20162 Milan, Italy

**Keywords:** Gait stability, Treadmill walking, Daily activity, Older adults

## Abstract

**Background:**

Gait variability can be considered an indirect measure of gait stability, in particular regarding temporal or spatial variability assessment. Physical activity, such as walking, is advised for the elderly and can be improved by gait stability. The aim of this study was to investigate the associations between gait stability and physical activity in women of different age ranges.

**Methods:**

Forty-two healthy women of different age ranges (18-40 yrs. and 65-75 yrs.) were recruited in the study. To assess physical activity, the subjects wore a multi-sensor activity monitor for a whole week, inferring the time spent in moderate to vigorous physical activity (MVPA). MVPA were analysed in bouts of at least 10 subsequent minutes (MVPA_bouts_) and in overall minutes (MVPA_tot_). A kinematic analysis was performed with an optoelectronic system to calculate gait variability - expressed as standard deviation (SD) and coefficient of variability (CV) of step width, stride length, stance and swing time (during treadmill walking at different speeds).

**Results:**

Elderly women, with high walking speed (5 km/h), and moderate step width variability (CV = 8–27%), met the recommended levels of physical activity (MVPA_tot_ and MVPA_bouts_). Furthermore, gait variability, adjusted for age and number of falls, was significantly and negatively associated with MVPA_tot_ only at 3.5 km/h, and with MVPA_bouts_ only at 4 km/h.

**Conclusions:**

In a population of healthy elderly women, gait variability was significantly and negatively associated with the level of physical activity. Healthy elderly women, with moderate gait variability (step width variability), and high preferred walking speed, seem to be able to meet the recommended levels of physical activity.

## Background

With age, structural and functional deterioration occurs in most physiological systems, despite the absence of suspected diseases. The term “elderly” applies to individuals aged 65 yrs. or older [1].

Regular physical activity, including aerobic exercise and muscle-strengthening activity, is essential for healthy ageing. Regular physical activity increases average life expectancy, because of its positive influence on chronic disease development, as it reduces age-related biological changes and their associated effects on health and well-being, and maintains functional capacity [2]. Remarkable evidence shows how physical activity reduces the risk of falls and injuries from falls, prevents or mitigates functional limitations, and is an effective therapy for many chronic diseases [3]. Older populations are generally less physically active than younger adults [2]. Although older adults may spend the same amount of time per day in exercise and lifestyle physical activities as younger normally active adults do, the most popular types of physical activities among older adults are significantly less demanding (walking, gardening, golf, low impact aerobic activities) than the ones performed by young adults [2].

Walking is an easily performable and a healthy form of physical activity which can be carried out at light, moderate or vigorous intensity. Walking is a convenient and daily type of exercise, requiring a significant amount of metabolic energy. Furthermore, it is the most commonly reported activity in adults who meet physical recommendations [4]. The public health benefits of walking go beyond its direct physiological benefits. Walking is often a group activity that results in social interaction, which also has effects on health as indicated by evidence revealing that low social interaction is associated with increased mortality [5]. Walking is a complex motor task generally performed automatically by healthy adults; however, walking is often no longer performed automatically by the elderly. Significant changes occur in gait across the life span, particularly after the age of 70. When walking, older adults require more attention concerning motor control than younger adults. Walking can result in falls, often with serious consequences. Gait impairments are one of the greatest risk factors for falls [6, 7]. Therefore, it is essential to identify individuals with an unstable gait in order to provide preventive and effective strategies.

Variability can be considered an indirect measure of gait stability, in particular with regard to variability of temporal or spatial measures [8]. Variability tends to increase with age [9, 10] and has been related to future impaired mobility [11]. Increased gait variability was further associated with many factors that are related to fall risks, such as strength, balance, and gait, but also with vitality, mental status, and quality of life. Variability was also strongly correlated with self-reported and performance-based measures of functional status [10].

Previous studies on walking in elderly adults have mainly focused on factors that influence variability. Egerton et al. [12] have recently tried to determine if temporal-spatial gait characteristics are associated with free-living ambulatory physical activity in relatively-healthy older people. Elhadi et al. [13] hypothesised that some biomechanical factors might contribute to lack of walking in older adults. However, only a small number of studies have examined the association between gait stability and the level of physical activity maintained by general older population.

### Aim of the study

The aim of this study was to compare treadmill walking at different speeds in younger and older women, in order to understand whether there is an association between gait parameters, and, particularly, gait stability, and physical activity levels (PAL).

## Methods

### Participants

Twenty-one young women and twenty-one older women (22.6±2.9 yrs.; 68.3±3.3 yrs., respectively) were recruited in the study. Informed consent was obtained from all participants enrolled in the study. The inclusion criteria were: young women aged between 18 to 40, and older women aged between 65 to 75, physically healthy and presenting no medical conditions that could prevent carrying out functional assessments or activities of daily living. Exclusion criteria included any current history of acute or chronic diseases or illnesses that would influence the regular outcome of the study. Anthropometric data are reported in Table 1.

**Table 1:** Anthropometric measurements in Older Adults and Younger Adults

	Older Adults		Young Adults				
Measurements	M	SD	M	SD	*p*-value	Power	ES
Height (m)	1.57	.07	1.62	.07	.0139	.713	−.7
Weight (kg)	64.5	9.1	59.1	5.9	.0287	.595	.7
BMI (kg/m^2^)	26.1	3.0	22.5	2.6	.0001	.992	1.3
WC (cm)	87.2	9.4	72.0	6.1	<.0001	1.000	2.0
HC (cm)	98.7	9.4	93.0	5.5	.0213	.646	.8
WHR (cm/cm)	.88	.05	.77	.05	<.0001	1.000	1.2
WHtR (cm/cm)	.56	.06	.44	.04	<.0001	1.000	2.4

Data are presented as mean (M), standard deviation (SD); *p*-value and power (one-way ANOVA); effect size (ES)

*BMI* body mass index, *WC* waist circumference, *HC* hip circumference, *WHR* waist to hip ratio, *WHtR* waist to height ratio

### Study design

An observational study was conducted for three days to ensure sufficient rest between trials. The research included a medical screening on the first day, as well as a quality of life questionnaire, in addition to anthropometric measurements, a maximal cardiorespiratory test and the evaluation of preferred walking speed. After one week, the resting metabolic rate was evaluated and participants wore an activity monitor for one week. After seven days, gait parameters and energy cost of walking were collected.

### Materials and procedure

Before starting the project, each subject underwent a complete medical examination; anthropometric measurements and number of falls in the previous year were recorded. Each subject completed a generic 36-item quality of life questionnaire, the Short Form 36v2 (SF-36v2). This questionnaire included 36 items grouped into eight scales: physical functioning (PF), bodily pain (BP), role-physical (RP), general health (GH), vitality (VT), social functioning (SF), role-emotional (RE) and mental health (MH). A score ranging from 0 to 100 was calculated for each scale. The Physical Component Summary (PCS) and the Mental Component Summary (MCS) were global scores obtained from the above mentioned eight scales by using calculation algorithms provided by the authors [14].

The resting metabolic rate (RMR) was measured through indirect calorimetry (Fitmate, Cosmed, Italy). A 15-min test was conducted, with the first discarded 5-min. The test site was physically comfortable, and participants rested between 10 and 20 min before being assessed [15]. The average value of the last 10 min was considered for further analysis. The RMR was expressed in kcal/day and was then converted into MJ/day.

The sleep heart rate (SHR) was evaluated with the activity monitor used to gauge physical activity during the free-living observation period, and was calculated as the highest value of the 30 lowest minute-by-minute heart rate readings during the 24-h period. At least three nights were taken into consideration [16, 17].

Preferred walking speed (PWS) was assessed with Polifemo Radio Light and Racetime 2 (Microgate, Italy) equipped with two photocells and a chronometer. Subjects were instructed to walk three times along a linear 14 m section at a comfortable walking pace. The PWS was calculated as the average time to walk the middle 10 m of 14 m [18, 19].

Maximal oxygen consumption (VO_2max_) was evaluated during a modified Balke treadmill test [20] to exhaustion, with breath-by-breath indirect calorimetry (Quark CPET, Cosmed, Italy). Before the test, all the subjects performed a warm up of 12 min, 2 min for each speed from 3 km/h to 5.5 km/h, increased by 0.5 km/h. During the modified Balke treadmill test, an established speed was maintained (4 km/h for older adults and 5.5 km/h for young adults); the test was started by setting the inclination at 0%; the inclination was subsequently increased by 2% after 1 min and by 1% every minute thereafter. In order to calculate the VO_2max_, all data were reduced to 30 s averages, and the mean value of the last minute of the test was taken into consideration. Maximal heart rate (HR_max_) was considered as the highest value at the end of the test.

Assessment of gait variability via biomechanical measures of foot kinematics provides a viable option for a quantitative evaluation of gait stability. Participants’ gait was recorded at 120 Hz with a 9-camera three-dimensional optoelectronic motion capture system (BTS Spa, Milano, Italy), calibrated according to the manufacturer’s guidelines before the trials. Twenty-three body landmarks (right and left tragion, acromion, olecranum, radius styloid process, anterosuperior iliac spines, great trochanter, femoral lateral epicondyle, lateral malleolus, heel, toe, glabella, spinous process of the 7th cervical vertebra, sacrum) were positioned on each participant by the same expert operator to reduce variability. Three additional markers were positioned on the treadmill base. The protocol comprised 1 min standing on the treadmill: in this phase, the subject was captured in a standing position for 5 s to provide reference for orthostatic position. Each subject had to walk uninterruptedly for 6 min without any support on a motor driven treadmill (TMX425C, Trackmaster, Cosmed, Italy) at six different speeds (3.0 - 3.5 - 4.0 - 4.5 - 5.0 - 5.5 km/h) resting 5 min between speeds. This rest period was added to allow metabolic values to reach basal conditions. Gait parameters and energetic measures were collected simultaneously. A portable breath-by-breath gas analysis system (K4b^2^, Cosmed, Italy) was used to store data, later downloaded and analysed with appropriate software. After filtering data (6 point smoothing), mean oxygen consumption (VO_2_) and heart rate (HR) were calculated for every speed considering the average 3 min from the 3rd to the 6th minute. In order to analyse the individual HR/VO_2_ correlation, HR values were plotted on VO_2_ values. Regarding biomechanical acquisitions, gait cycles were captured for 30 s, from the 3rd to the 4th minute of each speed test. Marker coordinates were tracked based on a previously created biomechanical model. Customised software within Matlab (The MathWorks Inc., Natick, MA, USA) was developed for data processing. Marker coordinates were filtered with a 15 Hz, low-pass 2nd order Butterworth filter. Each gait cycle (GC) was time-normalised to a standard 100 value sequence. Standard gait parameters (e.g. stride length, step width and stance/swing phase duration) were computed. Mean stride length and width, and stance and swing time for each individual were determined from all strides (right and left). Only values on the right side were considered as they did not statistically differ from those on the left one (Student’s t-test). Magnitude of variability was computed using both standard deviation (SD) - assessing the magnitude of stride length and width deviations - and stance and swing time with respect to the corresponding mean value, and coefficient of variation (CV), the percentage of the standard deviation compared to the mean: [(SD/mean) × 100].

To provide an accurate estimation of PAL during free-living activities, the Actiheart (AH, CamNtech, UK) was worn for seven whole and consecutive days. The participants were requested to carry on with their routine lifestyle while wearing the activity monitor. The instrument is minimally invasive and able to combine HR and movement monitor signals (ACC). The AH has been validated in adult individuals and in older adults [21]. Before the recording, a short signal test was performed to check HR signal integrity and to prevent artefacts due to noise. Once the signal test was successfully completed, the AH was set to long-term recording with epoch length of 1-min. At least 3 weekdays and 1 weekend day, with 10 or more consecutive hours of awake time, should be collected with good ACC and HR signals to represent a “typical week” [22]. AH can assess activity energy expenditure in addition to duration and intensity of physical activity [23, 24]. The present study took into consideration only ACC and HR combined values (Branched model) [24]. For every variable, the averaged value of all the days with available data was computed. To assess PAL, the research considered time spent in sedentary (SED, <1.5 METs), light (LIGHT, 1.5–2.9 METs) or moderate (MPA 3–6 METs) to vigorous (VPA >6 METs) physical activity [25]. In order to express the minutes spent in sedentary intensity, the minutes of sleep were subtracted from the numerical value of minutes spent at an intensity of <1.5 METs provided by the AH software. MPA and VPA were summed to obtain the total amount of time that participants spent in moderate and vigorous physical activity (MVPA ≥3 METs). Since PA guidelines [3] recommend to accumulate MVPA in bouts of at least 10 min, MVPA was also analysed in bouts of at least 10 consecutive minutes (MVPA_bouts_). Physical activity intensity was assessed through the AH using individual calibration, as suggested by Rennie et al. [26]. Individual calibration was completed by recording the following in the AH software: resting metabolic rate, sleep heart rate, maximal oxygen consumption, maximal heart rate, HR/VO_2_ relationship.

### Statistical analysis

Statistical analysis was carried out with the commercial software package STATVIEW 5.0. Normal distribution was verified and data were presented as the mean ± standard deviation. Statistical significance was set at *p* < .05.

Differences between younger adults (YA) and older adults (OA) were evaluated using a one-way ANOVA, with height as a covariate in stride length analysis. Two-way ANOVA was used to assess any significant differences in measured parameters 1) between groups (OA vs YA), 2) among speeds, and 3) interaction between the two subject groups and the different speeds. If the ANOVA indicated significant differences, the post-hoc Bonferroni method was used to perform multiple pair-wise comparisons between subject groups and test speeds. A one-way ANOVA was applied to analyse differences between MVPA_tot_ and MVPA_bouts_. Agreement between MVPA (tot or bouts) and ACSM guidelines (an average of 30 min per day) was computed using the One-Sample Sign Test. Cohen’s d effect size (ES) was also determined.

The Pearson correlation test was used to study the correlation between gait variables and physical activity outcome measures. The first Principal component (1PC) was used to summarise data of gait SD and CV of stride length, step width, stance and swing time, given that gait variability data (SD or CV) are linked to a unique latent variable. Two backward stepwise regression analysis models were employed to examine the associations between 1PC (CV) and MVPA_tot_ or MVPA_bouts_. The first model studied the bivariate association between CV and MVPA_tot_ and CV and MVPA_bouts_ (Unadjusted model); the second controlled for age and number of falls (Adjusted model).

## Results

### Differences between older adults and young adults

As expected, statistically significant (*p* < .05) differences, with a medium, large and huge effect size, were detected between the two groups regarding mean height, weight, body mass index (BMI), waist circumference (WC), hip circumference (HC), waist to hip ratio (WHR) and waist to height ratio (WHtR) (see Table 1). All subjects were classified as having a healthy weight [27].

No significant differences in MCS and PCS scores were found between groups; nevertheless, YA obtained a significantly higher (*p* < .001) PF score, a subscale of PCS, than OA. Both VO_2max_ and HR_max_ were significantly higher (*p* < .001), with a huge effect size, in YA compared to OA. The number of falls was significantly different (*p* < .05) between the two groups, with a medium effect size. No statistical differences were observed between groups in terms of preferred walking speed, sleep heart rate and resting metabolic rate (Table 2). Older women, with high preferred walking speed (5 km/h), met the recommended levels of physical activity (MVPA_tot_ and MVPA_bouts_).

**Table 2:** Preferred walking speed, quality of life and physiological measurements in Older Adults and Younger Adults

	Older Adults		Young Adults				
Measurements	M	SD	M	SD	*p*-value	Power	ES
MCS	48.4	9.7	47.1	7.2	.6232	.076	.2
PCS	53.3	6.8	56.9	5.4	.0664	.438	−.6
PCS (PF)	52.9	4.9	56.8	1.1	.0009	.956	−1.1
PWS (km/h)	4.8	0.5	5.0	0.7	.2461	.198	−.4
Falls (n)	.5	1.0	.0	.0	.0250	.619	.7
VO_2max_ (ml/kg/min)	26.9	5.1	39.3	4.7	<.0001	1.000	−2.6
HR_max_ (bpm)	162.3	6.4	194.7	8.1	<.0001	1.000	−4.6
RMR (MJ/die)	5.5	.9	5.9	1.2	.2046	.229	−.4
SHR (bpm)	59.4	4.8	58.1	8.3	.5294	.093	.2

Data are presented as mean (M), standard deviation (SD); p-value and power (one-way ANOVA); effect size (ES)

*MCS* mental component summary, *PCS* physical component summary, *PF* physical function, *PWS* preferred walking speed, *VO*_*2max*_ maximal oxygen uptake, *HR*_*max*_ maximal heart rate, *RMR* resting metabolic rate, *SHR* sleeping heart rate

Table 3 shows the minutes of physical activity, in particular time spent in SED, LIGHT, MOD, VIG, MVPA_tot_ and MVPA_bouts_, for OA and YA. MVPA_tot_ was significantly (*p* = 0.0026) higher than MVPA_bouts_. Only the time spent in VIG physical activity was significantly higher (*p* < .05), with a large effect size, for YA when compared with OA.

**Table 3:** Sedentary and physical activity behavior

	Older Adults		Young Adults				
Measurements	M	SD	M	SD	*p*-value	Power	ES
SED (min)	685.9	116.8	730.3	111.7	.2150	.221	−.4
LIGHT (min)	223.5	104.0	201.2	67.1	.4143	.122	.3
MOD (min)	61.2	63.4	55.0	46.4	.7179	.064	.1
VIG (min)	.2	.7	5.8	10.3	.0166	.686	−.8
MVPA_tot_ (min)	61.4	63.7	60.8	52.2	.9727	.050	.01
MVPA_bouts_ (min)	29.4	41.7	28.0	29.3	.8950	.052	.04

Data are presented as mean (M), standard deviation (SD); *p*-value and power (one-way ANOVA); effect size (ES)

*SED* sedentary time, *LIGHT* light physical activity, *MOD* moderate physical activity, *VIG* vigorous physical activity, *MVPA*_*tot*_ moderate and vigorous physical activity total, *MVPA*_*bouts*_ moderate and vigorous physical activity in bouts

**Fig. 1:**
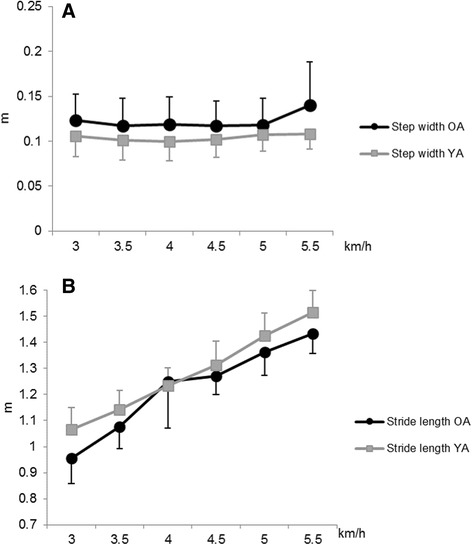
Step width (**a**) and stride length (**b**) at different walking speeds (mean + SD)

A statistical difference was found in step width (*p* < .05), with small and medium effect size, between the age groups. Stride length was significantly higher (*p* < .05), with medium, large and huge effect size, in YA than in OA (Fig. 1).

**Fig. 2:**
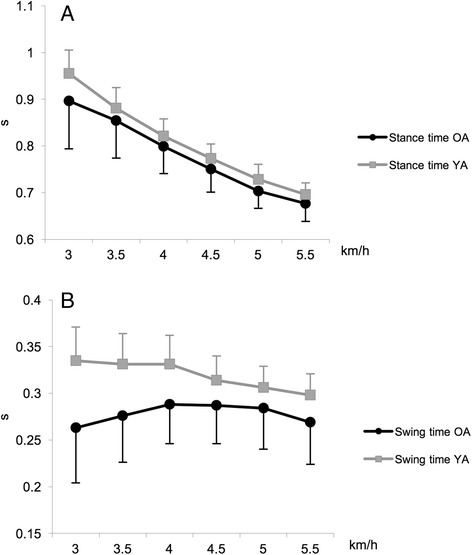
Stance (**a**) and swing (**b**) time at different walking speeds (mean + SD)

Swing and stance time measured in relation to the six speeds are shown in Fig. 2. Swing and stance time were significantly higher (*p* < .05), with a small, medium, large and huge effect size, in YA than in OA. Walking speed variability, expressed in terms of SD and CV, of the OA was not significantly different from the YA.

### Speed differences

Step width did not significantly differ between speeds, while stride length significantly increased with speed (*p* < .05) (Fig. 1). Stance time significantly decreased with the increasing of speed (*p* < .0001), while swing time did not significantly increase for any speed (Fig. 2).

**Fig. 3:**
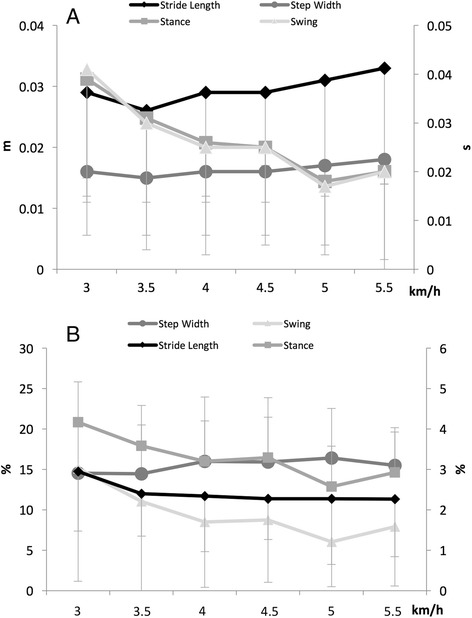
Gait variability expressed as SD (**a**) and CV (**b**) (mean + SD)

Significant differences in stance and swing time standard deviations were found among speeds (*p* < .05). Stride length variability, step width variability, stance variability and swing variability, identified as (SD) ± standard deviation, for all speeds, are illustrated in Fig. 3a.

Stride length, stance and swing time coefficients of variation were significantly (*p* < .001) influenced by speed. Stride length variability, step width variability, stance variability and swing variability, expressed as (CV) ± standard deviation, for all speeds, are shown in Fig. 3b.

With respect to CV, step width variability appeared to be larger than all the other gait parameters. Low step width variability can be considered by CV < 8% (lowest 5% of data); moderate step width variability can be measured by CV = 8–27% (middle 90% of data); high step width variability can be considered by CV > 27% (highest 5% of the data). Older women, with moderate step width variability, met the recommended levels of physical activity (MVPA_tot_ and MVPA_bouts_).

### Regression summary

A significant, moderate and positive correlation was found between preferred walking speed and both MVPA_tot_ (*r* = .324, *p* = .0360) and MVPA_bouts_ (*r* = .376, *p* = .0135), whereas no significant correlations were found between any gait parameters (stride length, step width, stance and swing time) and physical activity level. Significant, moderate and negative correlations were found between 1PC (CV) and both MVPA_tot_ (*p* < .05) and MVPA_bouts_ (p < .05) for each speed, whereas no significant correlations were found either between 1PC (SD) and MVPA_tot_ or between 1PC (SD) and MVPA_bouts_. For this reason, only 1PC (CV) was considered in gait variability measures. 1PC (CV) was significantly (*p* < .05) and negatively associated with MVPA_tot_ at all speeds, while 1PC (CV) was significantly (*p* < .05) and negatively associated with MVPA_bouts_ only at 3.5, 4, 4.5, 5 km/h (Unadjusted model). 1PC (CV) remained a significant (*p* < .05) indicator of MVPA_tot_ also after adjusting for age, and number of falls, only at 3.5 km/h and of MVPA_bouts_ only at 4 km/h (Adjusted model). In all models, 1PC (CV) clarified less than 20% of the variance (Table 4).

**Table 4:** Backward stepwise regression analysis between 1PC (CV) and MVPA_tot_ and MVPA_bouts_

	Model 1 Unadjusted						Model 2 Adjusted					
	MVPA_tot_			MVPA_bouts_			MVPA_tot_			MVPA_bouts_		
Speed	β	R^2^	*p*	β	R^2^	*p*	β	R^2^	*p*	β	R^2^	*p*
3	−3.289	.106	.0356^a^	−1.789	.082	.0664	−3.355	.142	.1158	−1.839	.127	.1560
3.5	−4.345	.154	.0102^a^	−2.333	.116	.0274^a^	−4.325	.187	.0470^a^	−2.322	.157	.0871
4	−3.758	.120	.0246^a^	−2.730	.166	.0075^a^	−3.547	.139	.1233	−2.594	.189	.0452^a^
4.5	−3.793	.116	.0272^a^	−2.184	.101	.0406^a^	−3.523	.131	.1458	−1.979	.121	.1732
5	−5.269	.152	.0108^a^	−2.648	.100	.0411^a^	−4.965	.159	.0830	−2.374	.117	.1888
5.5	−4.162	.110	.0319^a^	−2.176	.079^a^	.0722	−3.892	.119	.1808	−1.921	.096	.2740

^a^Statistically significant

In the two models all subjects were analysed together (YA and OA)

## Discussion

In this research, treadmill walking at different speeds in two groups of younger and older adults was assessed in terms of gait parameters and, particularly, of gait stability in order to study its association with physical activity levels.

The main findings of this research were that, (1) OA were as active as YA, despite being less suitable for cardiorespiratory fitness; (2) VIG physical activity was significantly higher in YA than in OA; (3) MVPA_tot_ was significantly higher than MVPA_bouts_; (4) OA presented a different walking pattern than YA, although age related differences in walking variability were not significant; (5) gait stability, expressed as CV, influenced the level of physical activity maintained, when expressed as MVPA_tot_ and MVPA_bouts_, for both OA and YA.

Physical activity in older adults is crucial for the prevention of diseases, sustaining independence and improved quality of life. Maintaining sufficient physical activity levels in older people is an important goal. Therefore, it is essential to identify gait characteristics of people at risk of future decline of PAL, or to identify possible interventions in order to help older people remain sufficiently physically active [12].

Direct measures of physical activity are generally considered more accurate, are not prone to response and recall biases, and are often used to validate indirect measures of physical activity [28]. Accelerometry yields an objective measurement of physical activity, and has been applied in large population-based studies of adults and older people monitoring overall physical activity, intensity-specific physical activity and time spent as sedentary time [29]. The Actiheart was developed to overcome the limitations of using solely HR or ACC data to predict physical activity. The addition of a physiological variable (HR) should provide a more precise evaluation of physical activity than using accelerometry data only, with respect to a wide range of activities [30]. Variability of conventional spatio-temporal parameters, such as standard deviation and coefficient of variation, offers a viable method for a quantitative evaluation of gait stability. The kinematic analysis system is one of the most specific and accurate methods to study gait variability [8, 9].

The study populations were composed of healthy and physically-active young and older adults, with high preferred walking speed and a good quality of life. Most individuals (52% of the elderly and 62% of the adults) reached recommended levels of physical activity if MVPA_tot_ are considered. Moreover, only 24% of the elderly achieved recommended levels of physical activity, compared to 43% of the adults, if MVPA_bouts_ are taken into account. In this case, most subjects failed to accumulate the health recommendations of 30 min of MVPA on 5 or more days per week. A closer focus on the range of moderate intensity activity achieved by older vs. younger adults may be useful, particularly in light of the debate about the extent of intensive activity beneficial to older adults [31]. There were no significant differences between groups regarding the minutes spent in sedentary activity, light activity and moderate activity. Older adults performed significantly less vigorous activities than the younger counterparts. In particular, no significant differences were detected between the time spent in MVPA when accumulated in bouts of at least 10 consecutive minutes, or when overall minutes of MVPA were considered. The absence of difference between age groups in overall activity is consistent with the finding of another study [32]. Furthermore, young and older adults showed similar preferred walking speeds, in accordance with Kang & Dingwell [33]. The average PWS of the elderly in this study is high when compared with literature data collected from women aged 65–75 yrs. [34]. In addition, according to the Almeida et al. classification [35] (in which “fallers” are those having suffered two or more falls in the previous year and “nonfallers” those having suffered either no falls or only one fall in the previous year), all young adults and 18 older adults can be classified as “nonfallers”, while only 3 older adults were classified as a “faller”.

The present results support the previous findings of a stride length impairment in elderly people [36], showing a decrease in stride length in older populations at almost all speeds. Indeed, in the present study, stride length ranges from 1.07 to 1.52 m in younger populations and from 0.96 to 1.43 m in older populations, respectively. A slowing of gait is also commonly reported in elderly individuals. According to Daley & Spinks [36], older adults over 67 years of age spend significantly longer time in the stance phase and significantly less time in the swing phase than younger adults. The present results do not confirm these data, showing that young adults spend more time in both stance and swing phases compared to elderly people. Presumably, our subjects did not need to modify their stance phase in order to obtain increased postural stability; as a matter of fact, they are still able to avoid a decline in speed of walking, typical of elderly.

Gait variability has become an important indicator in assessing human motor performance. According to the literature, the treadmill was used to collect a high number of consecutive gait cycles to investigate gait variability [37]. Furthermore, Owings et al. suggested that, compared to variability of spatial and temporal step kinematics, treadmill walking may be an acceptable representation of overground walking [38]. Increased gait variability is a risk factor for falls in older adults. In previous studies, a greater variability has been found in older adults for stride length [33] or step width [38, 39], regardless of differences in speed. Our results do not support these findings, because walking speed variability in older adults did not significantly differ from that of younger adults. Our data are consistent with those reported by Grabiner et al. [39] demonstrating that walking speed conditions influenced gait variables variability. Step width variability was larger than stride length variability when considering CV. In agreement with the literature, the present results suggest that for healthy young and older adults, gait variability of spatial parameters is a more important indicator of locomotion control than gait variability of temporal parameters, and step width variability is the most sensitive descriptor of locomotion control [38]. Our cut-off values are very close to the classification of Brach et al.: low step width variability (step width variability CV < 7%; lowest 5% of sample), moderate step width variability (step width variability CV = 7–30%; middle 90% of sample), and high step width variability (step width variability CV > 30%; highest 5% of the sample). However, Brach et al. found that extreme step width variability (i.e. either too much or too little) was associated with a history of falls in older adults, walking at, or close to, normal walking speed [40].

Hence, it would be interesting to study the impact of gait stability on physical activity participation. Egerton et al. [12] found that shorter step length, shorter step time, shorter swing time and higher cadence were associated with lower activity. Therefore, their results did not support the view that a worsening gait, analysing gait asymmetry, caused a decline in the level of physical activity maintained by rather healthy older people. They could not evaluate gait variability, recommending examining the relationship between gait variability and physical activity in future research. Our study showed that gait variability (CV), at different speeds (3.5 or 4 km/h), was significantly and negatively associated with the level of physical activity (MVPA_tot_ and MVPA_bouts_). Our models explained only 20% of the variance, suggesting that other variables influencing physical activity have not been taken into consideration.

The limitations of the study include: 1) the small sample size; 2) a poor population heterogeneity; 3) the absence of men in the sample selected; 4) self-selected women (selection bias); 5) only ‘young old’ elderly (65-75 yrs.). Our findings cannot unfortunately be generalised across the general older adult population, yet they may be useful with respect to healthy women aged between 65 and 75. Gait performance varies after the age of 75 and, in particular, after 80 [41], and it differs between men and women at the highest speeds [42]. Future research is required for a better understanding of the association between gait stability and the level of physical activity in a wide population range, including men and less healthy and older women.

## Conclusions

In a population of healthy elderly women (65-75 yr), gait variability was significantly and negatively associated with the level of physical activity. Healthy older women, with moderate gait variability (step width variability), and high preferred walking speed, seemed to meet the recommended levels of physical activity. As a practical application, these findings should be taken into account in the design of interventions aimed to improve overall activity.

## Abbreviations

ACC: Movement monitor signals
AH: Actiheart
BMI: Body mass index
BP: Bodily pain
CV: Coefficient of variation
GC: Gait cycle
GH: General health
HC: Hip circumference
HR: Heart rate
HR_max_: Maximal heart rate
MCS: Mental component summary
MET: Metabolic equivalent of task
MH: Mental health
MPA: Moderate physical activity
MVPA: Moderate and vigorous physical activity
MVPA_bouts_: Moderate and vigorous physical activity bouts
MVPA_tot_: Moderate and vigorous physical activity total
OA: Older adults
PAL: Physical activity level
PC: Principal component
PCS: Physical component summary
PF: Physical functioning
PWS: Preferred walking speed
RE: Role-emotional
RMR: Resting metabolic rate
RP: Role-physical
SED: Sedentary
SF: Social functioning
SF-36v2: Short Form 36v2
SHR: Sleeping heart rate
VO_2_: Oxygen consumption
VO_2max_: Maximal oxygen consumption
VPA: Vigorous physical activity
VT: Vitality
WC: Waist circumference
WHR: Waist to hip ratio
WHtR: Waist to height ratio
YA: Young adults
